# A miR-125/Sirtuin-7 pathway drives the pro-calcific potential of myeloid cells in diabetic vascular disease

**DOI:** 10.1007/s00125-022-05733-2

**Published:** 2022-06-16

**Authors:** Saula Vigili de Kreutzenberg, Alessandra Giannella, Giulio Ceolotto, Elisabetta Faggin, Roberta Cappellari, Marta Mazzucato, Chiara Fraccaro, Giuseppe Tarantini, Angelo Avogaro, Gian Paolo Fadini

**Affiliations:** 1 Department of Medicine – DIMED, University of Padova, Padova, Italy; 2 Veneto Institute of Molecular Medicine, Padova, Italy; 3 Department of Cardiac, Thoracic, Vascular Sciences and Public Health, University of Padova, Padova, Italy

**Keywords:** Ageing, Atherosclerosis, Cardiovascular disease, Epigenetics

## Abstract

**Aims/hypothesis:**

Ectopic calcification is a typical feature of diabetic vascular disease and resembles an accelerated ageing phenotype. We previously found an excess of myeloid calcifying cells in diabetic individuals. We herein examined molecular and cellular pathways linking atherosclerotic calcification with calcification by myeloid cells in the diabetic milieu.

**Methods:**

We first examined the associations among coronary calcification, myeloid calcifying cell levels and mononuclear cell gene expression in a cross-sectional study of 87 participants with type 2 diabetes undergoing elective coronary angiography. Then, we undertook in vitro studies on mesenchymal stem cells and the THP-1 myeloid cell line to verify the causal relationships of the observed associations.

**Results:**

Coronary calcification was associated with 2.8-times-higher myeloid calcifying cell levels (*p=*0.037) and 50% elevated expression of the osteogenic gene *RUNX2* in mononuclear cells, whereas expression of Sirtuin-7 (SIRT7) was inversely correlated with calcification. In standard differentiation assays of mesenchymal stem cells, SIRT7 knockdown activated the osteogenic program and worsened calcification, especially in the presence of high (20 mmol/l) glucose. In the myeloid cell line THP-1, SIRT7 downregulation drove a pro-calcific phenotype, whereas SIRT7 overexpression prevented high-glucose-induced calcification. Through the Janus kinase (JAK)/signal transducer and activator of transcription (STAT) pathway, high glucose induced miR-125b-5p, which in turn targeted SIRT7 in myeloid cells and was directly associated with coronary calcification.

**Conclusions/interpretation:**

We describe a new pathway elicited by high glucose through the JAK/STAT cascade, involving regulation of SIRT7 by miR-125b-5p and driving calcification by myeloid cells. This pathway is associated with coronary calcification in diabetic individuals and may be a target against diabetic vascular disease.

**Data availability:**

RNA sequencing data are deposited in GEO (accession number GSE193510; https://www.ncbi.nlm.nih.gov/geo/query/acc.cgi?acc=GSE193510).

**Graphical abstract:**

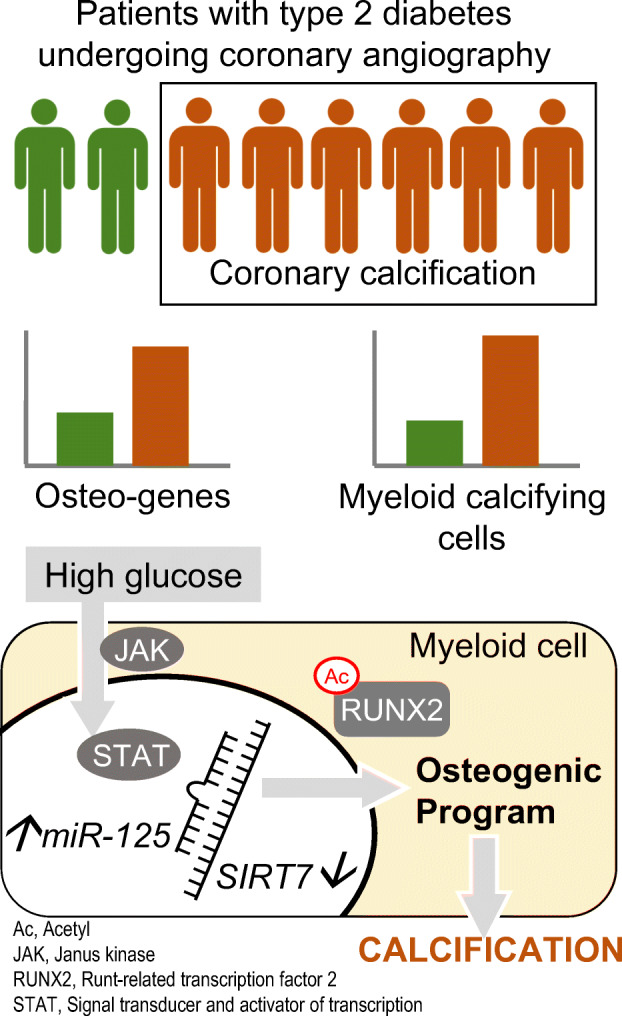

**Supplementary Information:**

The online version contains peer-reviewed but unedited supplementary material available at 10.1007/s00125-022-05733-2.

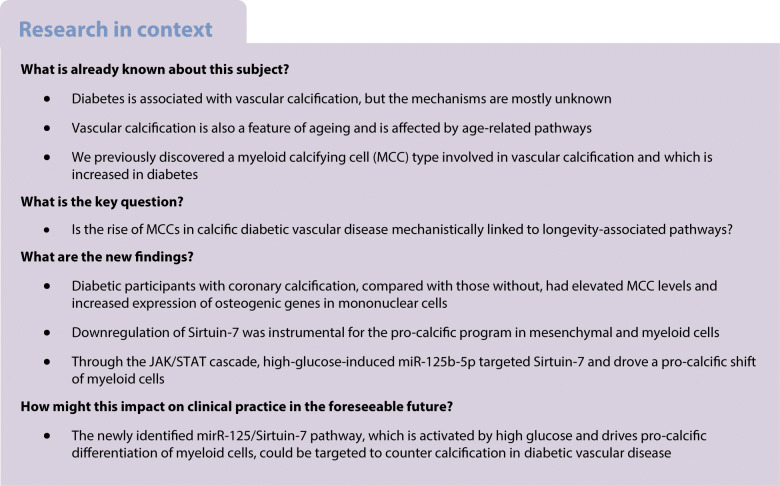



## Introduction

Diabetes accelerates atherosclerosis and is a major driver of cardiovascular disease. The atherosclerotic process observed in diabetes is more aggressive, diffuse and typically characterised by ectopic calcification [1]. Vascular calcification is a highly regulated process that often identifies a condition of elevated cardiovascular risk [2]. In individuals with or without type 2 diabetes, atherosclerotic coronary calcification (CC) is an independent predictor of major adverse cardiovascular events [3, 4].

Diabetic vascular calcification is linked to hyperglycaemia but extends beyond the effect of high glucose [5]. In vitro, osteogenic differentiation and mineralisation of vascular smooth muscle cells (VSMCs) from type 2 diabetes patients are accelerated due to the upregulation of genes associated with osteochondrogenesis [6, 7]. In addition to resident cells, circulating cells also play a major role in vascular calcification [8]. We previously described a mononuclear cell (MNC) subpopulation expressing osteocalcin (OC) and bone alkaline phosphatase (BAP) [9]. The emergence of OC^+^BAP^+^ monocytes was driven by the expression of *RUNX2,* the master gene regulator of osteogenesis [9]. These OC^+^BAP^+^ myeloid calcifying cells (MCCs) calcify in vitro and in vivo [9, 10]. In individuals with as compared with those without type 2 diabetes, MCCs were elevated in the bone marrow, blood and atherosclerotic plaques and could be reduced by glucose control [9]. However, the mechanisms generating excess MCCs in diabetes remain largely unknown. Since myeloid cells are prominently involved in atherosclerosis [11], drivers of the pro-calcific programs that switch MNCs to MCCs are of great interest to counter vascular calcification in diabetes.

Vascular calcification is highly prevalent, not only in diabetes but also in elderly people [12], due to several processes, including cellular senescence, oxidative stress and pro-calcific matrix changes. Among longevity-associated genes, sirtuins have been shown to participate in the calcification program and in cardiovascular disease in vitro [13, 14] and in vivo [15, 16]. Of note, diabetes is considered a disease of accelerated ageing, with premature end-organ damage and mortality [17]. Osteogenic differentiation drift, the release of pro-calcific micro-vesicles and accumulation of advanced glycation end-products are common mechanisms in diabetes- and ageing-induced vascular calcification [18, 19]. However, it is unclear whether the pathways driven by sirtuins are involved in MCC generation and diabetic vascular calcification.

We herein explored whether elevation of MCCs in diabetic vascular disease is associated with a pro-calcific program within myeloid cells that could be linked to the ageing-related pathway regulated by sirtuins.

## Methods

### Participants

The protocol was approved by the local ethics committee, and written informed consent was obtained from all participants. The study was conducted at the University Hospital of Padova between 2013 and 2015. We recruited type 2 diabetes patients aged 30–80 years, consecutively undergoing elective coronary angiography. Diabetes was defined according to American Diabetes Association criteria. Exclusion criteria were: type 1 diabetes; chronic kidney disease (defined as eGFR <45 ml min^−1^ 1.73 m^−2^); acute disease or infection; recent (<3 months) trauma, cardiovascular events or surgery; known osteoporosis or other bone-related disorders; recent (<6 months) fractures; immunological disorders or immunosuppressive therapy; and pregnancy or lactation. The indications for coronary angiography included: suspected/known ischaemic heart disease, valve disease and other specific conditions. The method for quantifying coronary calcium from coronary angiograms is described in the electronic supplementary material (ESM) Methods section.

For all participants, we recorded demographic characteristics, concomitant cardiovascular risk factors, laboratory examination results and pharmacological history. Smoking status was defined as current smoking of one or more cigarettes per day. Hypertension was defined as a systolic blood pressure >140 mmHg or a diastolic blood pressure >90 mmHg or the use of blood pressure-lowering drugs.

The morning preceding angiography, a fasting blood sample was obtained for biochemical analysis, flow cytometry and collection of peripheral blood mononuclear cells (PBMCs). HbA_1c_, total cholesterol, HDL-cholesterol, triacylglycerols, creatinine, uric acid concentrations, white blood cell counts and circulating levels of MCCs were determined. LDL-cholesterol and eGFR were calculated using Friedewald formula and Modified Diet in Renal Disease (MDRD) equations, respectively. Urine specimens were collected to measure AER.

We estimated that *n*=40 participants per group would be sufficient to detect a 0.8% difference in the frequency of MCCs in the monocyte gate with SD=1.3%, α=5% and power=80%.

### Flow cytometry

MCCs were identified and quantified by flow cytometry in participants' peripheral blood samples based on the surface expression of selected antigens. Briefly, after red cell lysis, 150 μl of blood was incubated with 10 μl of phycoerythrin (PE) anti-osteocalcin (OC clone 190125, R&D Systems, Minneapolis, USA, catalogue no. IC1419P) and 10 μl of allophycocyanin (APC) anti-bone specific alkaline phosphatase (BAP clone B4-78, R&D Systems, catalogue no. FAB1448A) [9].

### Gene expression

PBMCs were collected from 20 ml of heparinised blood over Histopaque-1077 (Sigma-Aldrich, Milano, Italy), and processed as previously described [16]. In PBMCs, the gene expression levels of Sirtuin-1 (SIRT1) to Sirtuin-7 (SIRT7), mammalian target of rapamycin (mTOR), p66shc, p53, forkhead box protein O (FOXO), Toll-like receptor 2 (TLR2), Toll-like receptor 4 (TLR4), runt-related transcription factor 2 (RUNX2), SP7 (also known as Osterix [OSX]) and NAMPT were determined by quantitative real-time PCR (qPCR). Methodological details are given in the ESM Methods section and ESM Table 1.

### Human telomerase reverse transcriptase expression

Human telomerase reverse transcriptase (hTERT) expression in PMBCs was determined by qPCR. More information can be found in the ESM Methods section.

### Telomere length

Mean telomere length was measured from DNA by qPCR, comparing telomere (T) repeat sequence copy number with the single-copy gene (S). More information can be found in the ESM Methods section.

### NAD activity

NAD^+^ was measured in PBMCs using the NAD^+^/NADH quantification assay kit, according to the protocol of the manufacturer (Biovision, Milpitas, CA, USA). The NAD^+^ content was measured from the standard curve and normalised to the protein content of the sample.

### Western blot analysis

Protein expression levels of SIRT-1, SIRT-7, RUNX2 and OSX were determined by western blot (WB) analysis. Antibodies against SIRT1 (catalogue no. 2496), SIRT7 (catalogue no. 5360), RUNX2 (catalogue no. 8486) and GAPDH (catalogue no. 2118) were purchased from Cell Signaling Technology (Euroclone, Milan, Italy), while the antibody against OSX (catalogue no. 229258) was from Abcam (Prodotti Gianni, Milan, Italy). RUNX2 lysine acetylation was analysed by immunoprecipitation of RUNX2 followed by WB using acetyl-lysine antibody (Cell Signaling Technology, catalogue no. 9814). Further details are provided in the ESM Methods section.

### Culture in osteogenic medium

To obtain human mesenchymal stromal/stem cells (hMSCs), bone marrow aspirate pellets were plated on tissue culture Petri dishes with mesenchymal medium. Then, 1×10^6^ hMSCs were cultured in osteogenic induction medium with Mesencult Osteogenic Stimulatory Kit (StemCell Technologies, Vancouver, QC, Canada); cells were maintained by the addition of fresh osteogenic induction medium every 2–3 days, over 21 days. At the end of the differentiation period, cells were used to determine biomarkers of calcification.

THP-1 cells were obtained from the American Type Culture Collection (Manassas, VA, USA). Cells were cultured in RPMI 1640 medium (Sigma-Aldrich) supplemented with 10% FBS, 1% l-glutamine and 1% antibiotic solution in a humid atmosphere containing 5% CO_2_ at 37°C. THP-1 cells (3×10^6^/cm^2^) were plated in six-well plates coated with Matrigel (Corning Incorporated Life Sciences, Tewksbury, MA, USA) for 3 weeks in the complete osteogenic medium. The medium was replaced after 1 week, and then every 3 days. At the end of the 3 weeks, cells were stained with von Kossa. Matrigel-extracted cells next underwent dispase digestion, and were then used for gene and protein expression, as previously described [20]. In separate experiments, hMSCs and THP-1 cells were cultured in osteogenic medium with high glucose (20 mmol/l) or mannitol (20 mmol/l, as osmotic control). In separate experiments, THP-1 cells grown in complete medium with normal or high glucose were treated with selective drugs: signal transducer and activator of transcription (STAT) 1 inhibitor Fludarabine (Aurogene, Rome, Italy, catalogue no. S1491), STAT3 inhibitor Stattic (Tocris, Bristol, UK, catalogue no. 2798) and Janus kinase (JAK) inhibitor AG-490 (Tocris, catalogue no. T3434) for 24 h at the final concentration of 10 μmol/l. ELISA was used to measure the release of paracrine factors, as described in the ESM Methods section.

### Von Kossa staining and calcium measure

To verify calcification in cell cultures, we performed the von Kossa stain in hMSCs and THP-1 osteogenic medium-differentiated cells. Calcium in cell cultures was measured using the HCl extraction method. More information can be found in the ESM Methods section.

### SIRT7 silencing and overexpression

hMSCs or THP-1 cells were cultured in six-well dishes with 100 nmol/l short interfering RNA (siRNA) against SIRT7 or scrambled siRNA (scr-SIRT7) for 48 h or during the osteogenic differentiation, with four doses of siRNA against SIRT7. Cells were then transfected with Lipofectamine 3000 (Invitrogen, Carlsbad, CA, USA). Efficiency of SIRT7 knockdown was determined by qPCR. Validated Silencer Select siRNA pre-designed sequences were purchased from Applied Biosystems (Waltham, MA, USA). For SIRT7 overexpression, THP-1 cells were transfected with a SIRT7 plasmid, pcDNA3.1 SIRT7flag (Addgene.org plasmid 13818, accessed on Feb 2022). The transfection of plasmid (3 μg/well) was carried out by Lipofectamine 3000 using a control vector (pcDNA3.1/NT-GFP, Invitrogen). SIRT7 knocking was verified by qPCR.

### Mimic-miR-125b-5p and antagomir

Synthetic mimic-miR-125b-5p (50 nmol/l) was transfected into THP-1 cells for 24 h, 48 h and 72 h using Lipofectamine 3000 (Invitrogen) [21]. At the end of each experiment, the numbers of live and total cells were counted with trypan blue staining. The cell viability was 80–90%. SIRT7 gene and protein expression levels were assessed by qPCR and WB, respectively, as described above. For miR-125b-5p inhibition, THP-1 cells were cultured in an osteogenic medium with high glucose (20 mmol/l) and in the presence or absence of antagomir-125b-5p. At the end of 3 weeks, cells were collected to determine SIRT7 and RUNX2, and for the quantitative detection of calcification. Experiments were performed in the presence of scrambled miR-125b-5p.

### Candidate microRNA selection and gene expression analysis

We mined the publicly available GEO DataSets to select microRNAs (miRNAs) regulated by osteogenic conditions and high glucose. RNA was extracted from THP-1 cells and candidate miRNAs quantified by qPCR. Further details are provided in the ESM Methods section.

### RNA sequencing analysis

The gene expression profile of osteogenic induced-THP-1 cells (osteo-THP-1) in high vs normal glucose was analysed by RNA sequencing (RNA-seq), as described in the ESM Methods section, and is deposited in GEO (GSE193510).

### Statistical analysis

Data are presented as mean ± SD in the text and Table 1, and as mean ± SEM in histograms with superimposed individual data points. Non-normally distributed variables were log-transformed before analysis with parametric tests. Comparison between two group means was performed using unpaired two-tailed Student’s *t* test. ANOVA with least square difference post hoc test was used to compare three or more groups. Differences between categorical data were assessed by χ^2^ test. Linear correlation between two continuous variables was explored with Pearson's *r* and age-adjusted using the partial correlation function. SPSS version 22.0 was used, and statistical significance was accepted at *p<*0.05.

**Table 1:** Participant characteristics

Variable	All (*n* = 87)	CC− (*n* = 18)	CC+ (*n* = 69)	*p*
Demographics				
Number	87	18	69	
Age (years)	68 ± 8	67 ± 12	68 ± 7	0.743
Sex (M/F)	70/17	12/6	58/11	0.574
Diabetes duration (years)	13.7 ± 9.9	13.5 ± 11.8	13.8 ± 9.3	0.936
Risk factors				
Hypertension (no/yes)	94.2	94.9	94.1	0.579
Smoking (no/active or previous)	66.7	55.6	69.6	0.275
Family history of CVD (no/yes)	73.6	77.8	72.5	0.770
BMI (kg/m^2^)	28.7 ± 4.3	29.4 ± 5	28.5 ± 4	0.416
Waist (cm)	105 ± 13	107 ± 17	105 ± 12	0.747
Laboratory exams				
HbA_1c_				0.276
mmol/mol	58 ± 12	54 ± 10	60 ± 13	
%	7.5 ± 1.6	7.1 ± 1.3	7.6 ± 1.6	
Total cholesterol				0.517
mmol/l	4.0 ± 0.9	3.9 ± 0.7	4.1 ± 0.9	
mg/dl	156 ± 34	151 ± 27	157 ± 36	
HDL-cholesterol				0.233
mmol/l	1.2 ± 0.3	1.3 ± 0.4	1.2 ± 0.3	
mg/dl	46 ± 12	49 ± 14	45 ± 11	
LDL-cholesterol				0.434
mmol/l	2.2 ± 0.8	2.1 ± 0.5	2.3 ± 0.9	
mg/dl	86 ± 31	81 ± 21	87 ± 33	
Triacylglycerols				0.811
mmol/l	1.4 ± 0.7	1.4 ± 0.6	1.4 ± 0.7	
mg/dl	125 ± 59	122 ± 55	126 ± 60	
Serum creatinine (μmol/l)	91.6 ± 27.6	89.2 ± 19.6	92.2 ± 29.6	0.680
eGFR (ml min^−1^ 1.73 m^−2^)	79.4 ± 23.8	77.5 ± 20.1	79.9 ± 24.6	0.707
AER (mg/g)	158 ± 627	19 ± 15	188 ± 689	0.423
Uric acid (mmol/l)	0.37 ± 0.13	0.40 ± 0.15	0.36 ± 0.11	0.195
White blood cells (×10^6^/l)	6.783 ± 2.485	6.214 ± 2.041	6.936 ± 2.584	0.277
Coronary artery disease				
Calcific vessels	1.8 ± 1.1	0 ± 0	2.3 ± 0.7	<0.001
Calcific segments	3.3 ± 2.4	0 ± 0	4.2 ± 1.9	<0.001
SYNTAX score	19 ± 16	6 ± 10	22 ± 16	<0.001
Therapies, %				
Insulin	32.9	30.8	33.3	0.861
Metformin	56.2	53.9	56.7	0.855
Incretin	11.0	15.3	10.0	0.579
Sulfonylurea	23.3	15.4	25.0	0.464
Pioglitazone	2.7	0	0.03	0.511
ACE inhibitors/ARBs	81.3	93.8	78.1	0.156
Calcium channel blockers	35.4	18.8	39.6	0.088
Beta blockers	55.0	43.8	57.8	0.381
Anti-platelet agents	77.5	62.5	81.4	0.178
Statin	73.8	68.8	75.0	0.617
Warfarin	15.0	12.5	15.6	0.758

The table shows demographic and anthropometric variables in the whole study group, and comparison between participants without (CC−) and with (CC+) CC. Data are presented as mean ± SD for continuous variables or as frequencies and percentages for categorical variables

ARBs, angiotensin receptor blocker; M/F, male/female

## Results

### CC associates with elevated MCCs and a pro-calcific ageing signature

**Fig. 1:**
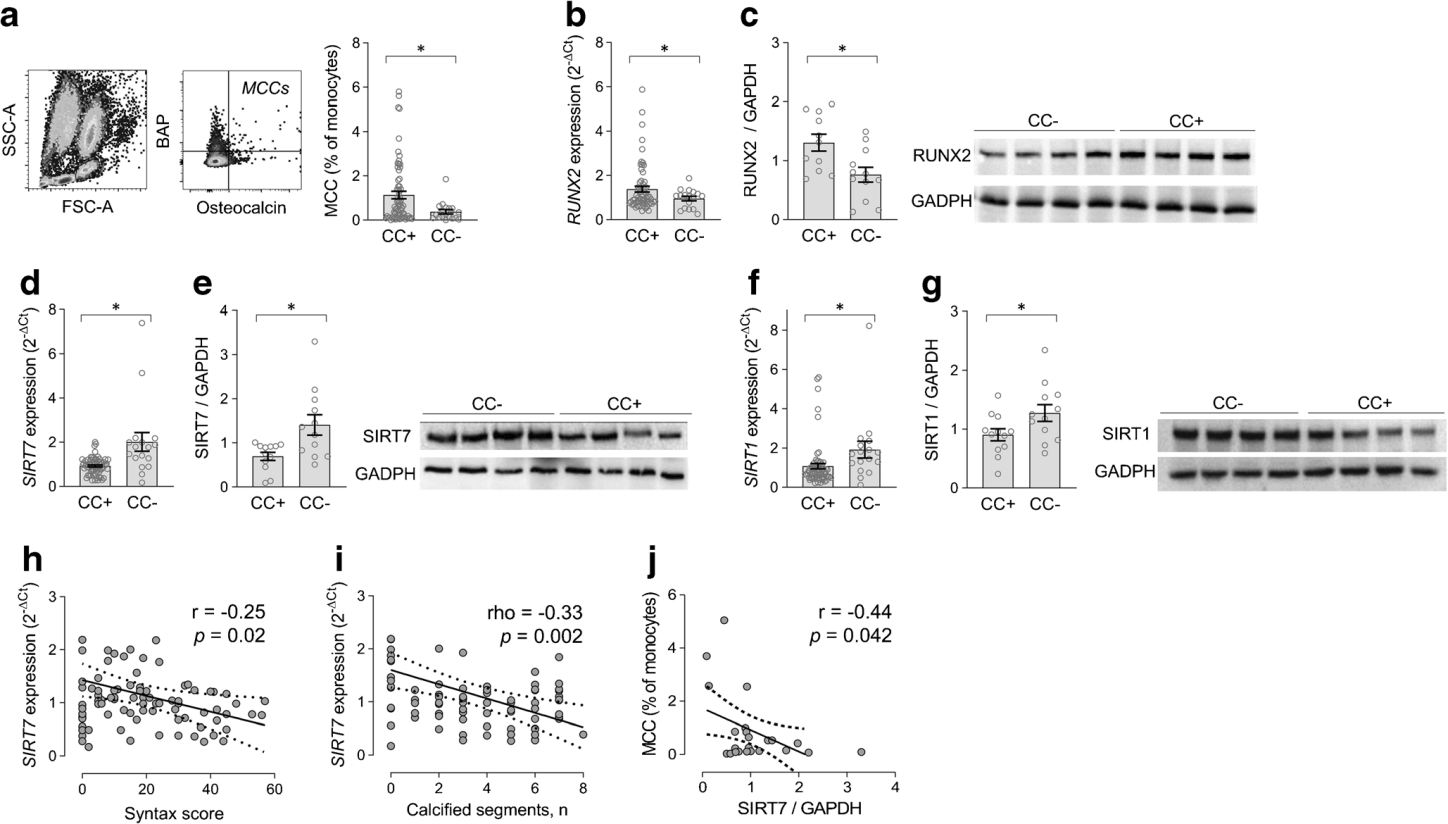
Findings from the clinical study cohort. (**a**) The gating strategy used to identify monocytes and MCCs in the upper-right corner of the OC/BAP plot. Levels of MCCs, expressed as percentage of the MNC gate, in participants with (CC+) and without (CC−) CC. (**b**, **c**) Expression of RUNX2 gene (**b**) and protein (**c**; relative to GAPDH, with representative WB images on the right) in MNCs from CC+ and CC−. (**d**–**g**) Expression levels of SIRT7 and SIRT1 genes and proteins in the two groups of participants are shown in (**d**) and (**e**), and (**f**) and (**g**), respectively, with representative WB images on the right-hand parts of (**e**) and (**g**). Protein expression was quantified in 12 randomly selected participants in each group. (**a**–**g**) Histograms indicating mean with SEM bars and superimposed circles indicating individual data points. *p<0.05 for the indicated comparison (exact *p* values are given in the text). (**h**, **i**) Linear correlations between SYNTAX score (**h**) or the number of coronary segments with calcification (**i**) and SIRT7 gene expression. (**j**) Linear correlation between SIRT7 protein content in MNCs and the level of MCCs. Correlation plots show the regression line with dashed 95% CIs, as well as the age-adjusted Pearson’s *r* and *p* values

Participant characteristics according to the presence or absence of CC are summarised in Table 1. No significant difference was observed between the two groups, allowing an unbiased comparison of molecular pathways. In line with our prior findings, circulating levels of MCCs were 2.8-times higher in diabetic participants with CC (*p=*0.037; Fig. 1a). One participant had an unusable sample for mRNA quantification. In the MNC population, which represents the MCC progeny, CC was associated with significantly 50% elevated expression of the master regulator of the cellular osteogenic program, RUNX2, at gene (1.38±1.06 vs 0.95±0.42; *p=*0.013; Fig. 1b) and protein (1.70±1.43 vs 0.76±0.44; *p=*0.041; Fig. 1c) level. Importantly, RUNX2 is inherently related to MCC differentiation from MNCs [9].

Since vascular calcification identifies an ageing phenotype, we examined the expression of a series of longevity-associated genes and protein products in MNCs (ESM Table 2). In participants with CC (CC+), as compared with those without (CC−), SIRT7 expression was reduced at gene (−45%; *p=*0.021; Fig. 1d) and protein (−25%; *p=*0.012; Fig. 1e) level. To a lesser extent, SIRT1 expression was also reduced (gene, −35%; *p=*0.017; protein, −14%; *p=*0.047; Fig. 1f, g) in CC+ vs CC− participants. Within the MNC fraction, we detected no difference in gene expression of *SIRT7* and *RUNX2* between lymphocytes and monocytes (ESM Fig. 1). After age-adjustment, *SIRT7* gene expression inversely correlated with SYNTAX (Synergy between percutaneous coronary intervention with TAXUS and cardiac surgery) score (a measure of coronary artery disease severity; *R*=−0.25; *p=*0.02; Fig. 1h), and with the number of calcified arteries (*R*=−0.38; *p<*0.001) and segments (ρ=−0.33; *p=*0.002; Fig. 1i). An inverse correlation between SIRT7 protein and MCC levels was also observed (*R*=−0.44; *p=*0.042; Fig. 1j). Such associations were not present for SIRT1 (not shown). As an additional pathway linked to ageing, we examined telomere length and telomerase expression, which were similar in CC+ vs CC− participants (ESM Fig. 2). We thus focused on SIRT7 to evaluate its role in calcification and correlation with MCCs.

### SIRT7 promotes the osteogenic program in mesenchymal cells

**Fig. 2:**
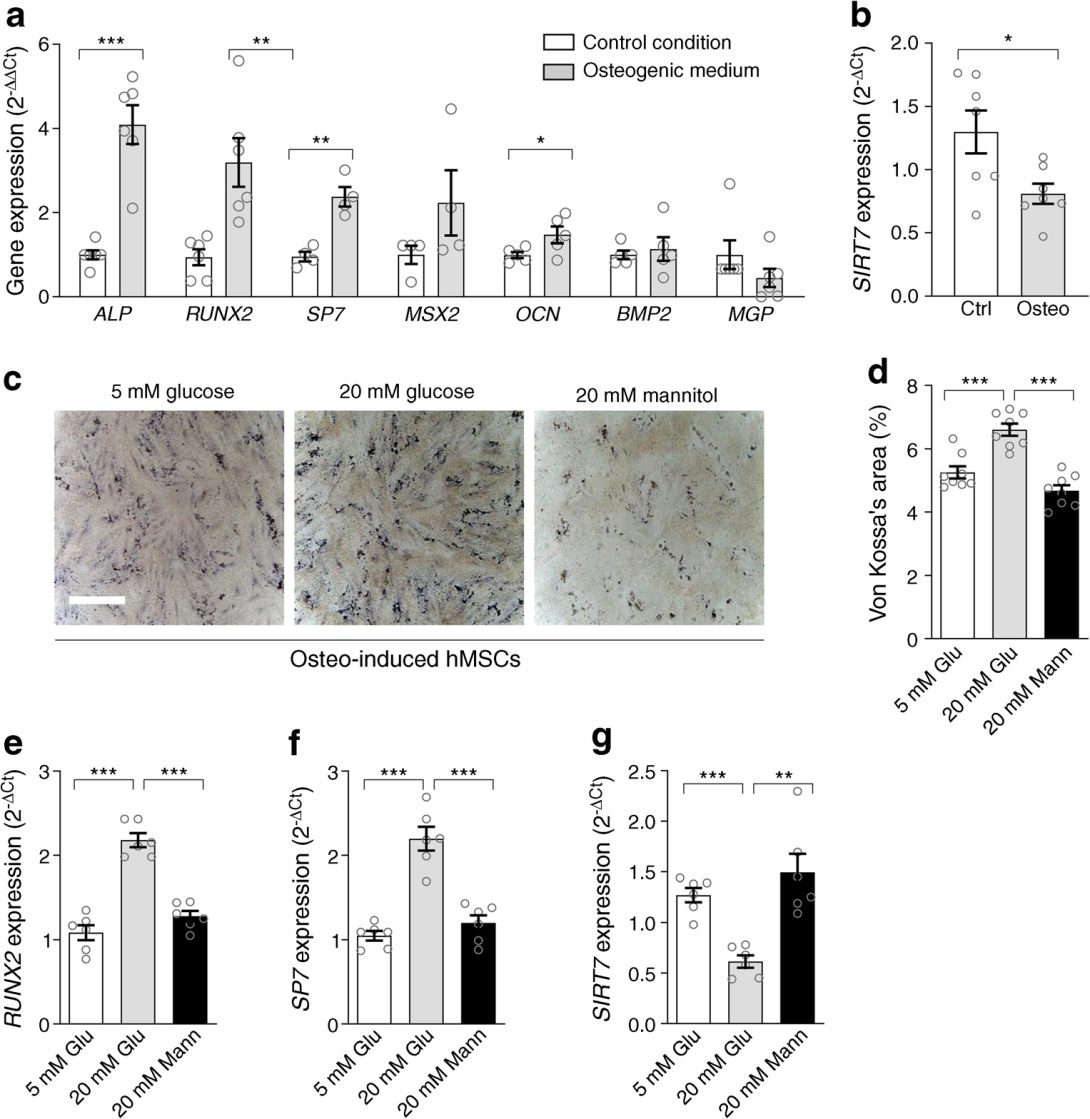
SIRT7 downregulation during pro-calcific differentiation and in high glucose. (**a**) Expression of selected pro-calcific genes during osteogenic differentiation of hMSCs compared with the control condition. (**b**) *SIRT7* gene expression in hMSCs grown in the osteogenic medium (Osteo) compared with the control unstimulated condition (Ctrl). (**c**) Representative microphotographs of von Kossa staining of hMSCs under osteogenic medium (osteo-induced) and grown in normal glucose (5 mmol/l), high glucose (20 mmol/l) or equimolar mannitol as osmotic control. (**d**) Quantification of von Kossa-stained area, as percentage of the total area, in the three conditions shown in panel (**c**). (**e**–**g**) Gene expression of *RUNX2* (**e**), *OSX/SP7* (**f**) and *SIRT7* (**g**) in osteo-induced hMSCs grown in 5 mmol/l glucose, 20 mmol/l glucose or 20 mmol/l mannitol. Histograms show means with SEM bars and superimposed circles indicate individual data points. For the indicated comparisons, statistical significance was as follows: *p<0.05; **p<0.01; ***p<0.001. Glu, glucose; Mann, mannitol

The exposure of hMSCs to the osteogenic medium induced typical genes of the osteogenic program (including *RUNX2*, *OSX/SP7* and *OCN*; Fig. 2a), concomitantly suppressing *SIRT7* expression (*p*=0.022; Fig. 2b). To simulate the diabetes milieu, we exposed hMSCs to normal glucose (5 mmol/l), high glucose (20 mmol/l) or equimolar mannitol (osmotic control) concentrations during osteogenic differentiation. Compared with normal glucose and osmotic controls, high glucose increased calcified area upon von Kossa staining (Fig. 2c, d). Osteogenic differentiation of hMSCs in the presence of high glucose further increased *RUNX2* (Fig. 2e) and *OSX/SP7* expression (Fig. 2f) and downregulated *SIRT7* (Fig. 2g).

**Fig. 3:**
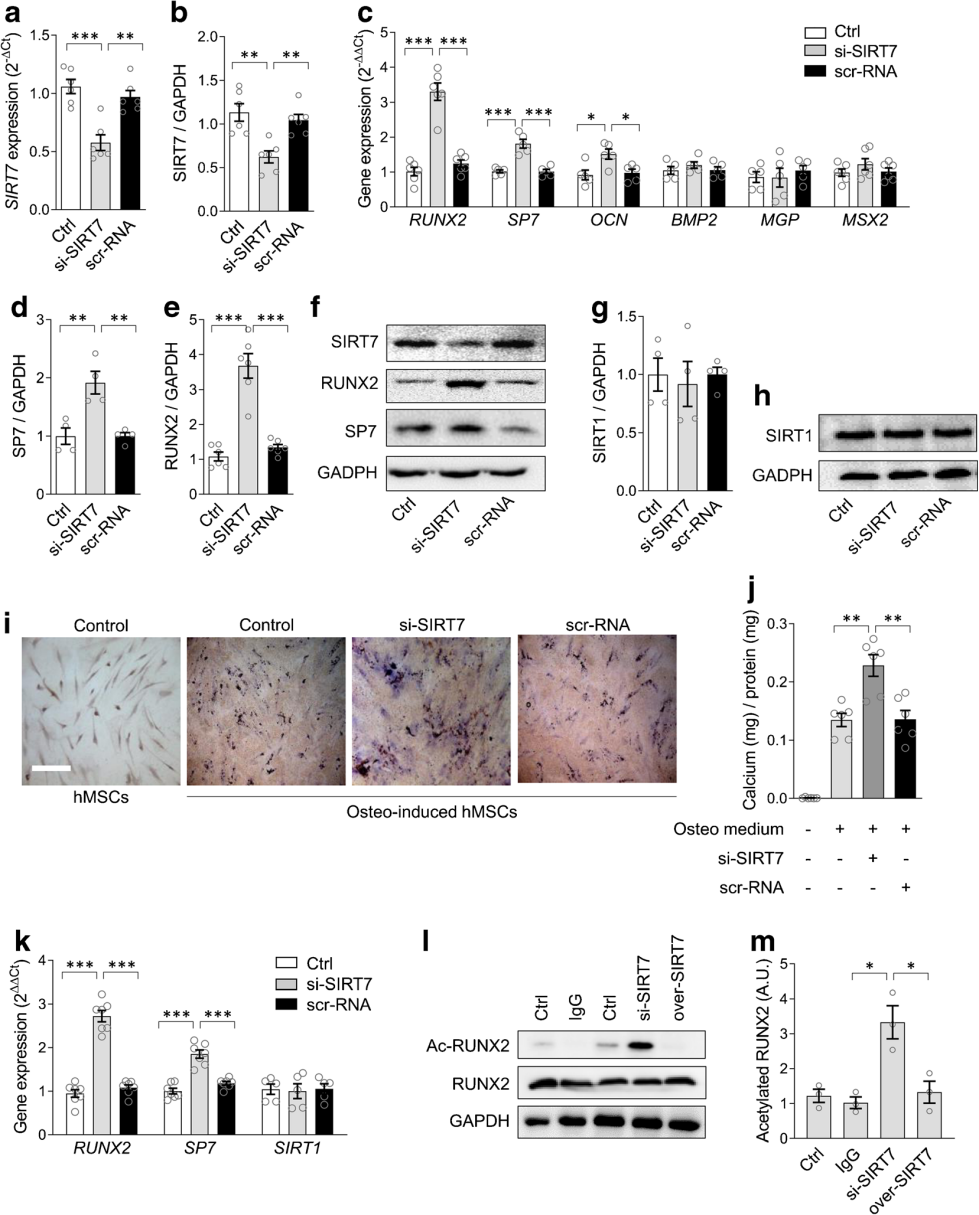
SIRT7 regulates the pro-calcific program. (**a**, **b**) Effects of *SIRT7* knockdown with siRNA (si-SIRT7) on SIRT7 expression at gene (**a**) and protein (**b**) level in hMSCs. (**c**) Effects of *SIRT7* knockdown by siRNA on gene expression of hMSCs grown in normal conditions. (**d**–**f**) Induction of OSX/SP7 (**d**) and RUNX2 (**e**) proteins by si-SIRT7; (**f**) representative WBs. (**g**, **h**) SIRT1 protein content in hMSCs with SIRT7 knockdown. (**i**) Representative microphotographs of hMSCs cultured under normal medium or osteogenic medium with or without si-SIRT7. (**j**) Quantification of calcium content in the culture assay shown in (**i**) using a chemical extraction method. (**k**) Changes in the expression of *RUNX2*, *OSX/SP7* and *SIRT1* in hMSCs grown in osteogenic medium with or without si-SIRT7. In all conditions from (**a**) to (**k**), two negative controls are shown: untreated control cells (Ctrl) and cells transfected with a scramble-RNA (scr-RNA). (**l**, **m**) Lysine-acetylated RUNX2 was quantified by WB (**l**) in hMSCs grown in osteogenic medium with or without SIRT7 silencing (si-SIRT7) or SIRT7 induction (over-SIRT7). The control IgG conditions are shown (**m**). Histograms show means with SEM bars and superimposed circles indicate replicate experiments. For the indicated comparisons, statistical significance was as follows: *p<0.05; **p<0.01; ***p<0.001. Ac-RUNX2, acetylated RUNX2; AU, arbitrary units

To verify whether *SIRT7* downregulation was a cause or effect of the osteogenic program, we silenced *SIRT7* expression in hMSCs. We achieved a ~50% reduction in *SIRT7* gene expression by siRNA (Fig. 3a, b). Interestingly, in *SIRT7*-knockdown cells, gene and protein expression of RUNX2 and OSX/SP7 were increased compared with control cells and with scramble-RNA-transfected cells (Fig. 3c–f). Gene expression of *OCN* was also increased, whereas *BMP2*, *MGP* and *MSX2* expression was unaffected (Fig. 3c). *SIRT1* expression remained unchanged in *SIRT7*-silenced cells (Fig. 3g, h), thereby ruling out unspecific effects and interactions between SIRT7 and SIRT1. These results suggested that *SIRT7* downregulation may drive the osteogenic program. Indeed, when we exposed hMSCs to the osteogenic medium in the presence of siRNA-SIRT7, greater calcium deposition was observed with von Kossa staining than in control or scramble-RNA osteogenic-induced hMSCs (Fig. 3i). Calcium measured by a quantitative assay confirmed that *SIRT7* silencing increased calcium deposition during osteo-induction of hMSCs (Fig. 3j). Consistently, gene expression of *RUNX2* and *OSX* was significantly increased in SIRT7-silenced osteo-mesenchymal stem cells (MSCs), whereas *SIRT1* expression did not change (Fig. 3k).

### RUNX2 is a target of SIRT7 deacetylase activity

Since SIRT7 acts as a protein lysine deacetylase, we examined whether SIRT7 regulates RUNX2 acetylation in hMSCs grown in osteogenic medium. We performed experiments in SIRT7-silenced and SIRT7-overexpressing cells and determined the level of RUNX2 lysine acetylation. The degree of RUNX2 acetylation induced by osteogenic differentiation of hMSCs was strongly increased in SIRT7-silenced cells (*p*=0.01 vs IgG control), while RUNX2 acetylation was abolished by SIRT7-overexpressing cells (Fig. 3l, m).

### SIRT7 regulates glucose-induced calcification by myeloid cells

**Fig. 4:**
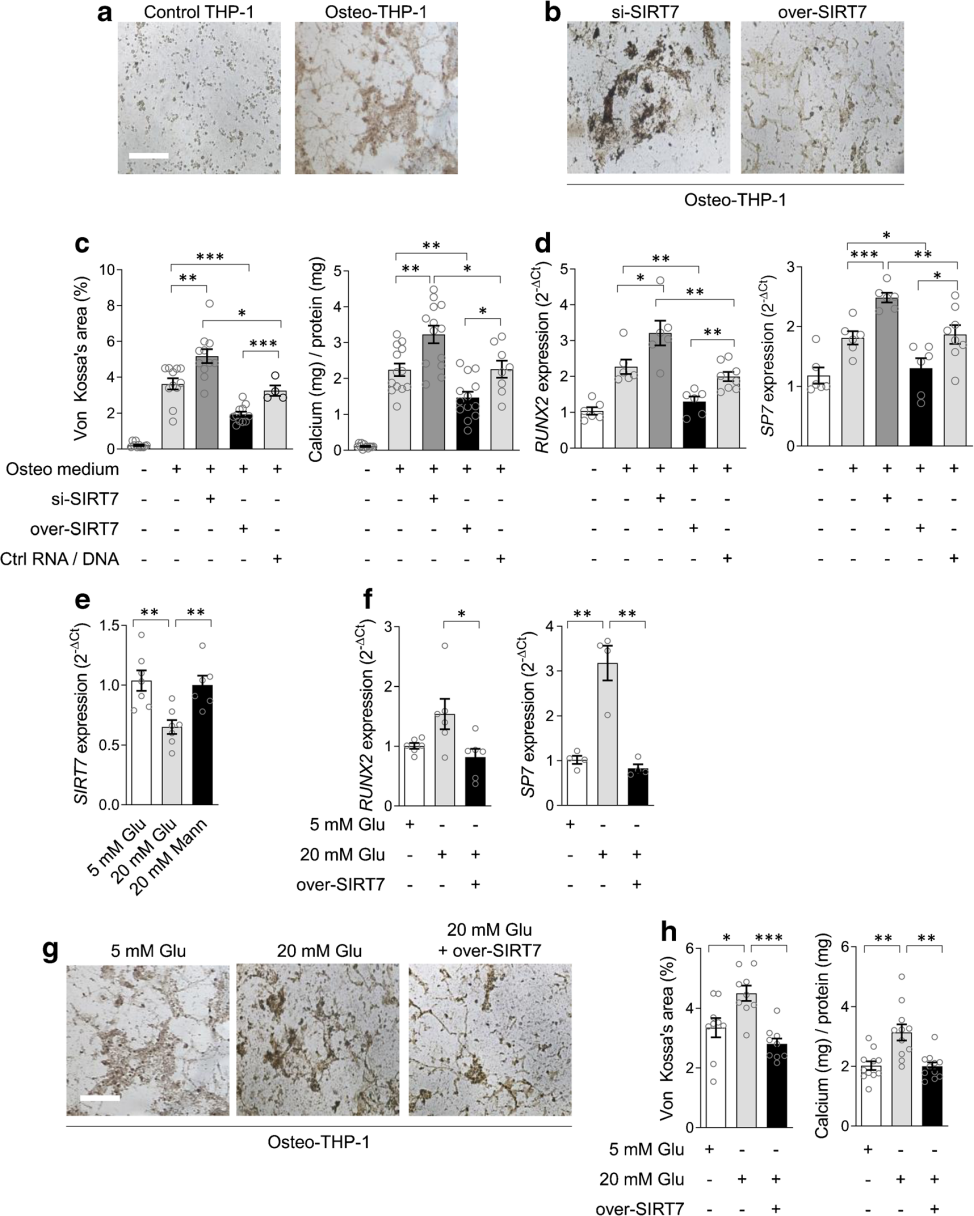
SIRT7 regulates myeloid cell calcification in high glucose. (**a**) Effects of incubating the myeloid cell line THP-1 cells with osteogenic medium on calcification assessed by von Kossa staining. (**b**) Calcification, as assessed by von Kossa staining, by THP-1 cells grown in osteogenic medium and transfected with siRNA against *SIRT7* or with a SIRT7 plasmid to obtain overexpression. (**c**) Quantification of von Kossa-stained area (left) and quantitative measure of calcium content (right) in the four culture conditions of THP-1 cells shown in panels (**a**) and (**b**). (**d**) Gene expression of *RUNX2* and *OSX*/*SP7* in the four conditions shown in (**a**) and (**b**). (**e**–**g**) Effects of exposing THP-1 cells to high (20 mmol/l) glucose (Glu) compared with normal glucose (5 mmol/l) and high mannitol (Mann) on gene expression of *SIRT7* (**e**), and pro-calcific genes *RUNX2* and *OSX*/*SP7* (**f**). (**g**) Representative microphotographs of THP-1 cells grown in osteogenic medium and exposed to normal (5 mmol/l) or high (20 mmol/l) glucose with or without overexpression of SIRT7 (over-SIRT7). (**h**) Quantification of calcification in the three conditions shown in (**g**) using von Kossa staining (left) and quantitative measure of calcium content (right). Histograms show means with SEM bars and superimposed circles indicate individual data points. For the indicated comparisons, statistical significance was as follows: *p<0.05; **p<0.01; ***p<0.001. Ctrl, control

We then moved to analysing whether SIRT7 regulates calcification by the human myeloid cell line THP-1. After verifying that THP-1 cells can be forced toward a pro-calcific phenotype resembling MCCs (Fig. 4a), we performed experiments in *SIRT7*-silenced and *SIRT7*-overexpressing THP-1 cells grown in osteogenic medium (Fig. 4b). Successful silencing and overexpression of SIRT7 are shown in ESM Fig. 3. In SIRT7-silenced osteo-THP-1 cells, the percentage area stained with von Kossa and the amount of calcium deposition were enhanced compared with osteo-THP-1 in the control condition (Fig. 4c). Consistently, in SIRT7-overexpressing cells, the percentage of von Kossa-stained area and calcium concentrations were reduced (Fig. 4c), as was gene expression of *RUNX2* and *OSX* (Fig. 4d). THP-1 cells grown in osteogenic medium secreted more calgranulin S100A8 (+25%; *p=*0.006) than THP-1 cells in the control medium, which was further enhanced by SIRT7 knockdown (+57%; *p=*0.007; ESM Fig. 4). In addition, SIRT7-silenced THP-1 cells secreted more allograft inflammatory factor-1 (AIF-1; +36%; *p=*0.006; ESM Fig. 4), which is a monocyte-derived factor implicated in atherosclerotic calcification [10].

Since high glucose promoted MSC calcification with concomitant *SIRT7* downregulation, we evaluated if SIRT7 overexpression prevented glucose-induced calcification by myeloid cells. As observed with hMSCs, incubation of osteo-THP-1 cells with high glucose significantly reduced *SIRT7* expression (Fig. 4e), induced *RUNX* and *OSX/SP7* (Fig. 4f), and increased calcified area measured by von Kossa staining and calcium content (Fig. 4g, h). Overexpressing *SIRT7* during osteogenic induction of THP-1 cells reduced calcified area and repressed the rise of *RUNX2* and *OSX* induced by high glucose (Fig. 4f, h).

Altogether, these data support a causative role for *SIRT7* downregulation in the pro-calcific differentiation of myeloid cells exposed to high glucose and provide a mechanistic explanation for the findings obtained in individuals with or without CC.

### SIRT7 downregulation under high glucose depends on miR-125b-5p

**Fig. 5:**
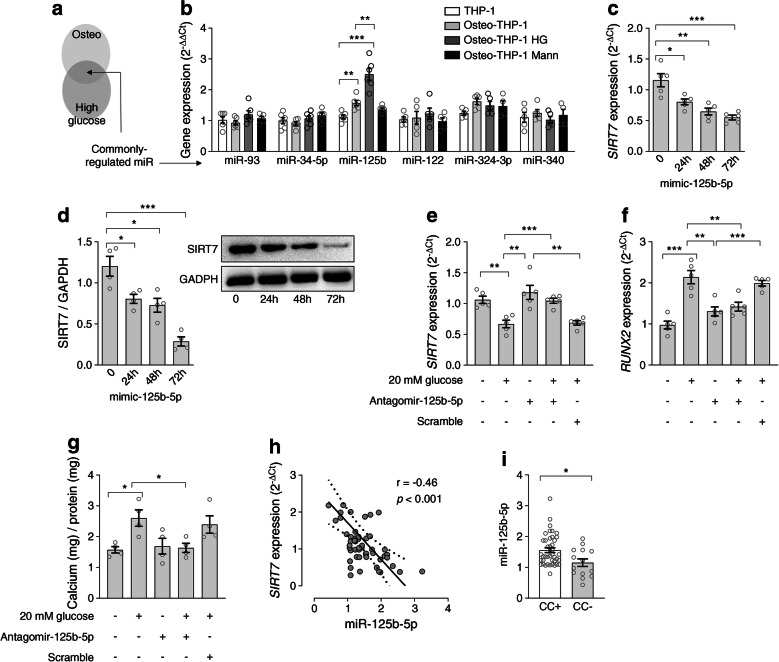
SIRT7 is controlled by miR-125b-5p in myeloid cells. (**a**) Strategy used to identify miRNAs (miR) commonly regulated by both exposure to osteogenic conditions (osteo) and exposure to high glucose. (**b**) Expression of candidate miR in unstimulated THP-1 cells, and osteo-THP-1 cells in normal glucose, high glucose (HG, 25 mmol/l) or equimolar mannitol (Mann). (**c**) Time course of the expression of *SIRT7* in THP-1 cells incubated with a miR-125b-5p mimic RNA. (**d**) Time course of SIRT7 protein content in THP-1 cells incubated with a miR-125b-5p mimic RNA (representative WBs of SIRT7 protein and of the housekeeping GAPDH before [0] and 24 h, 48 h and 72 h after incubation with miR-125 mimic are also shown). (**e**–**g**) Expression of *SIRT7* (**e**) and *RUNX2* (**f**) and calcium content (**g**) in osteo-THP-1 cells exposed to normal or high (20 mmol/l) glucose in the presence or absence of antagomir against miR-125b-5p or negative control (scramble). (**h**) Linear correlation between miR-125b-5p expression and *SIRT7* gene expression in MNCs of participants with diabetes undergoing coronary angiography (the regression line is shown, with dashed lines representing 95% CI). (**i**) Mean expression of miR-125b-5p in diabetic participants according to the presence (CC+) or absence (CC−) of CC. Histograms show means with SEM bars and superimposed circles indicate individual replicates. For the indicated comparisons, statistical significance was as follows: *p<0.05; **p<0.01; ***p<0.001

We explored mechanisms whereby high glucose reduced *SIRT7* expression, thus driving the pro-calcific switch of myeloid cells. *SIRT7* is post-transcriptionally regulated by several miRNAs [22], and we found that both osteogenic induction and high glucose downregulated *SIRT7* expression. We therefore mined GEO2R using specific search terms to identify candidate miRNAs consistently upregulated in human cells by both high-glucose and osteogenic conditions (Fig. 5a) or known to target *SIRT7*. We thus quantified the expression of miR-125b, miR-122, miR-93, miR-340, miR-34c-5p and miR-324-3p during osteo-differentiation of THP-1 cells in the presence of normal or high glucose or osmotic control (Fig. 5b). During osteo-differentiation of THP-1 cells, miR-125b was significantly upregulated in osteo-THP-1 cells under high glucose compared with normal glucose and osmotic control. The expression of other candidates remained unchanged (Fig. 5b). To determine whether miR-125b-5p affected *SIRT7* expression, we transfected mimic-miR-125b-5p into THP-1 cells for 24 h, 48 h and 72 h and examined SIRT7 gene (Fig. 5c) and protein (Fig. 5d) expression. miR-125b-5p mimic repressed *SIRT7* mRNA expression level at 24 h, with maximal effect on SIRT7 protein between 48 h and 72 h (Fig. 5c, d). To confirm that glucose-induced miR-125b-5p repressed *SIRT7*, enhancing the osteogenic phenotype, we cultured THP-1 cells under osteogenic conditions with antagomir-125b-5p in normal or high glucose. Antagomir-125b-5p restored gene expression of *SIRT7*, attenuated gene expression of *RUNX2* and ultimately reduced calcium content (Fig. 5e–g). These data show that high glucose induces miR-125b-5p, which, in turn, is responsible for *SIRT7* downregulation and potentiation of calcification. A significant inverse correlation further supporting this link was observed between plasma miR-125b-5p levels and *SIRT7* gene expression in MNCs of participants with type 2 diabetes (*r*=−0.46; *p<*0.001; Fig. 5h). In addition, MNC expression of miR-125b-5p was significantly higher in CC+ compared with CC− participants (*p*=0.01; Fig. 5i).

### The JAK/STAT pathway drives a pro-calcific switch under high glucose

**Fig. 6:**
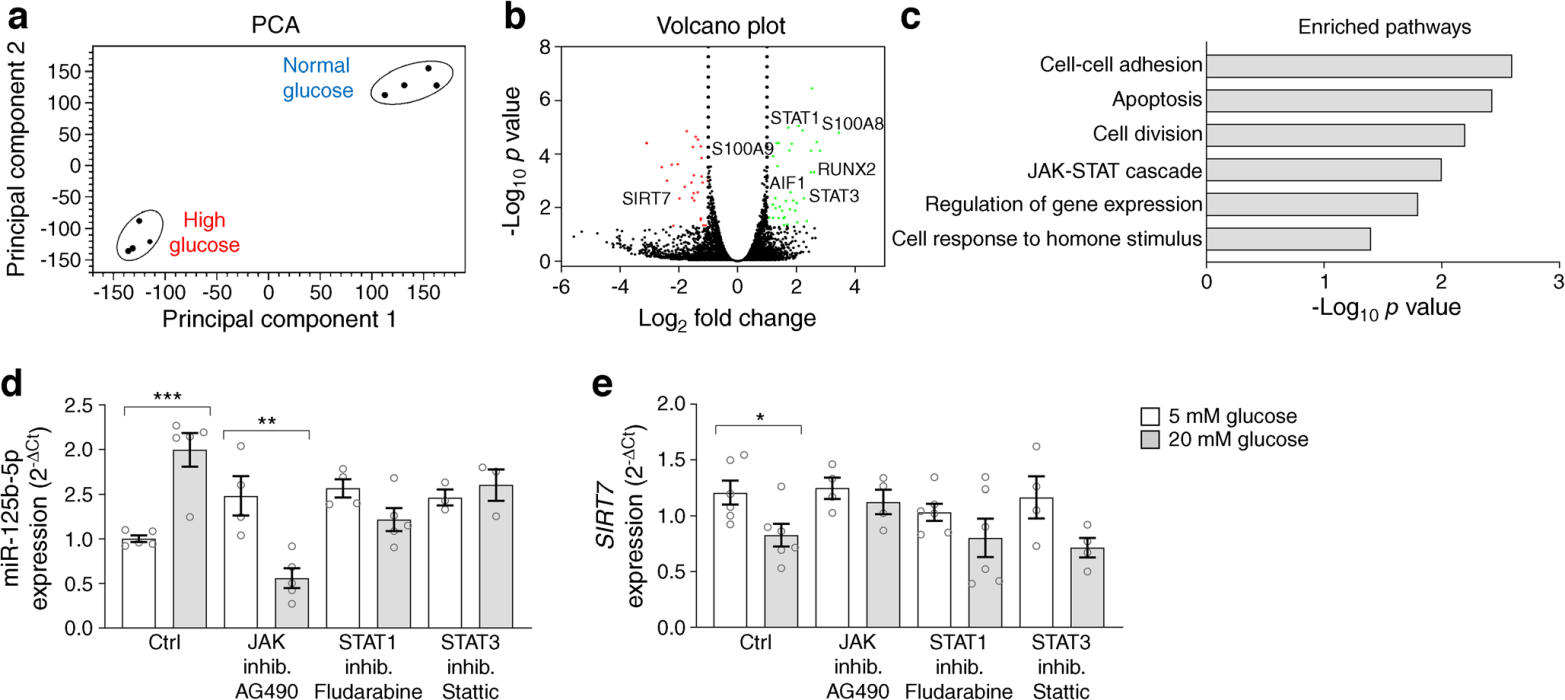
Pathways induced by high glucose in osteo-THP-1 cells. (**a**) PCA performed on gene expression profiles of osteo-THP-1 cells exposed to normal (5 mmol/l) or high (20 mmol/l) glucose. (**b**) Volcano plot showing genes upregulated or downregulated by high vs normal glucose in osteo-THP-1 cells. Labels indicate some key differentially regulated genes. (**c**) Pathway enrichment analysis performed with the DAVID platform to identify pathways and cellular functions based on differentially expressed genes in the high vs normal glucose condition. (**d**, **e**) miR-125b-5p (**d**) and *SIRT7* (**e**) expression in osteo-THP-1 cells cultured in normal (5 mmol/l, white) or high (20 mmol/l, grey) glucose concentration in the presence or absence of inhibitors of JAK, STAT1 and STAT3. Histograms show means with SEM bars and superimposed circles indicate individual replicates. For the indicated comparisons, statistical significance was as follows: *p<0.05; **p<0.01; ***p<0.001. Ctrl, control; Inhib., inhibition

We performed RNA-seq to investigate the transcriptional signature induced by high glucose in THP-1 cells grown in osteogenic conditions (GSE193510). The principal component analysis (PCA) displays a net separation of cells grown in normal or high glucose (Fig. 6a). Differential gene expression analysis identified 36 upregulated and 27 downregulated genes (Fig. 6b, ESM Table 3). Among upregulated genes, we identified genes encoding calcium-binding proteins (*S100A8*, *S100A9* and *AIF1*) and genes encoding transcription factors (*STAT1*, *STAT3*, *RUNX2* and *OSX*/*SP7*), while *SIRT7* was confirmed among downregulated genes. Based on gene ontology and pathway enrichment analysis, we found that high glucose enriched for genes involved in cell–cell adhesion, apoptosis, cell division, the JAK/STAT cascade, regulation of gene expression and cellular response to hormone stimulus (false discovery rate-corrected *p*<0.05; Fig. 6c). Based on these results, we focused on the JAK/STAT pathway as a candidate mechanism whereby high glucose drives the pro-calcific potential of THP-1 cells. We quantified miR-125b-5p and *SIRT7* expression in osteo-THP-1 cells under high glucose in the presence of JAK/STAT inhibitors. Inhibition of JAK with AG490 (10 μmol/l for 24 h) strongly reduced miR-125b-5p expression in high glucose, while inhibition of STAT1 or STAT3 attenuated miR-125b-5p induction by high glucose (Fig. 6d). Furthermore, in the presence of a JAK inhibitor, osteo-THP-1 cells in high glucose did not show downregulation of *SIRT7* expression as observed in the absence of JAK inhibitor (*p*=0.027 vs 5 mmol/l glucose; Fig. 6e). Therefore, the JAK/STAT cascade is likely to drive miR-125b-5p expression under high glucose and downstream *SIRT7* downregulation.

## Discussion

This translational study shows that high glucose induces miR-125b-5p, which targets SIRT7, thereby driving a pro-calcific shift of myeloid cells. Evidence for this pathway is supported by clinical data on type 2 diabetes patients undergoing coronary angiography and by robust in vitro findings. MCCs are generated from myeloid MNCs when they express high levels of RUNX2 and develop pro-calcific potential, a process that takes place in the bone marrow and possibly in target tissues, such as atherosclerotic plaques [9, 10]. We previously found that MCCs are elevated in the blood of type 2 diabetes patients and in endarterectomy specimens with large calcified areas [9]. We extend those findings by showing that MCCs were higher in type 2 diabetes patients with CCs than in those without.

To explore the molecular pathways driving such association within myeloid cells, we focused on longevity-associated genes because vascular calcification is also a prominent feature of ageing. Among a series of longevity-related pathways, SIRT1 and SIRT7 were downregulated in myeloid cells of individuals with type 2 diabetes and CCs, but only SIRT7 showed clear relationships with the extent of calcification and with MCC levels. SIRT7 is known to prevent osteogenic differentiation of hMSCs [23] and to regulate bone mass [24]. Before moving to myeloid cells, we tested this pathway in MSCs, representing the most straightforward model of osteo-induction. SIRT7 downregulation during osteogenesis was instrumental to activation of the hMSC pro-calcific program. In addition, we found that SIRT7 controls the acetylation state of RUNX2, which affects the pro-calcific program. De-acetylation of RUNX2 by SIRT7 would promote RUNX2 protein degradation [25]. *RUNX2* mRNA level was consistently regulated, possibly to avoid the accumulation of post-translationally modified protein, as RUNX2 binds to its own promoter via OSE2 (osteoblast-specific element-2) and controls its expression [26].

Interestingly, elevated glucose concentrations similar to those seen in decompensated diabetes or during daily hyperglycaemic spikes were able to suppress SIRT7 to the same extent as did osteogenic conditions. We then investigated the role of SIRT7 in myeloid cells using THP-1 cells. This human myelo-monocytic cell line has been validated as a model to study the role of monocytes in the vasculature [27, 28], and is particularly suitable for gene expression manipulation. THP-1 cells grow in suspension but can be induced toward a pro-calcific phenotype that resembles MCCs due to the myeloid origin, the elevated expression of RUNX2 and other MCC markers, as well as the pattern of calcification observed in vitro. We found that SIRT7 regulates calcification also in THP-1 cells, suggesting that this is a highly conserved pathway not limited to cells physiologically prone to osteogenic differentiation like hMSCs. Silencing and overexpressing *SIRT7* in THP-1 cells efficiently modulated calcification induced by osteogenic medium. Of note, *SIRT7* overexpression suppressed the pro-calcific program induced by high glucose in myeloid cells. This result makes a striking parallel to our findings in type 2 diabetes patients with CC, who displayed elevated MCC levels and suppression of SIRT7, along with activation of the pro-calcific program (*RUNX2* and *OSX/SP7*) within myeloid cells. We previously reported that MCCs exert their pro-calcific potential at least in part through secretion of calcium-binding proteins, including S100A8/9 and AIF-1 [10]. We herein confirm that THP-1 cells cultured under osteogenic conditions secrete more S100A8, which, along with secretion of AIF-1, was potentiated by knockdown of *SIRT7*. Since AIF-1 stimulates VSMC mineralisation and myeloid-selective AIF-1 overexpression in atherosclerosis-prone *ApoE*^−/−^ mice increases atherosclerotic calcification [10], we speculate that AIF-1 is a crucial driver of the pro-calcific potential of myeloid cells.

It has been recognised that many of the molecular pathways altered by hyperglycaemia are driven by epigenetic reprogramming [29, 30]. Among epigenetic regulators, diabetes not only affects sirtuins, but also impacts the cellular repertoire of miRNAs [31, 32]. We therefore examined if and how changes in miRNA expression accounted for the high-glucose-induced SIRT7 downregulation. Having shown that high glucose and osteogenic stimuli similarly reduced SIRT7 expression, we screened candidate miRNAs based on their common regulation in both conditions and identified miR-125b-5p. Prior studies defined SIRT7 as a target of miR-125 [33–36]. Here, we extend those findings, showing that high glucose markedly induced miR-125 and that miR-125 alone was able to mimic the effects of high glucose on SIRT7 downregulation. Importantly, such findings obtained in THP-1 cells were confirmed by the inverse correlation observed between miR-125 and SIRT7 expression in participants’ MNCs. Analysis of gene expression patterns of osteo-THP-1 cells in high glucose identified several differentially expressed genes belonging to the pathway we have herein described, including *SIRT7*, *RUNX2*, *OSX/SP7*, *S100A8/9* and *AIF1*. Interestingly, based on pathway analysis of RNA-seq data, the JAK/STAT cascade was enriched in THP-1 cells exposed to high glucose during osteogenic differentiation. This enabled us to demonstrate that high-glucose-induced miR-125b-5p upregulation could be prevented by inhibitors of the JAK/STAT pathway, which also rescued *SIRT7* levels.

Some limitations of our study need to be acknowledged. We analysed gene expression within the MNC fraction in humans, composed of lymphocytes and monocytes. Though we detected similar levels of expression of *SIRT7*, *RUNX2* and miR-125b-5p between lymphocytes and monocytes, the newly discovered pathway is supposed to be involved in calcification by myeloid, not lymphoid, cells. Furthermore, we have not yet tested the miR-125b-5p/SIRT7 pathway in a relevant in vivo animal model. Based on our data, we speculate that myeloid-specific overexpression of miR-125b-5p would aggravate calcification in a mouse model of atherosclerosis. Vice versa, an atherosclerosis-prone inducible myeloid-specific miR-125b-5p knockout model will be needed to firmly demonstrate the therapeutic potential of tackling this newly uncovered pathway. Finally, whether our findings can be generalised to non-diabetic vascular disease is uncertain and deserves future studies.

In summary, we show that high glucose induces miR-125b-5p, possibly via JAK/STAT, which in turn suppresses SIRT7 expression, dysregulating RUNX2 acetylation, and thus driving a pro-calcific program in myeloid cells. Along with the higher MCC levels, this pathway is exacerbated in type 2 diabetes patients with CC, as evidenced by the elevated MNC miR-125b-5p expression. Therefore, our study uncovers a new pathway that could be exploited to tackle calcification as one prototypical feature of diabetic vascular disease.

## Supplementary information

ESM 1 (PDF 180 kb)

## Data Availability

RNA sequencing data are deposited in GEO (accession number GSE193510; https://www.ncbi.nlm.nih.gov/geo/query/acc.cgi?acc=GSE193510). Original data are available from the corresponding author on reasonable request.

## Abbreviations

AIF-1: Allograft inflammatory factor 1
BAP: Bone alkaline phosphatase
CC: Coronary calcification
hMSC: Human mesenchymal stromal/stem cell
JAK: Janus kinase
MCC: Myeloid calcifying cell
miRNA: microRNA
MNC: Mononuclear cell
MSC: Mesenchymal stem cell
OC: Osteocalcin
osteo-THP-1: Osteogenic induced-THP-1 cells
OSX: Osterix
PBMC: Peripheral blood mononuclear cell
PCA: Principal component analysis
qPCR: Quantitative real-time PCR
RNA-seq: RNA sequencing
RUNX2: Runt-related transcription factor 2
siRNA: Short interfering RNA
SIRT1: Sirtuin-1
SIRT7: Sirtuin-7
SP7: Transcription factor Sp7, also known as osterix (OSX)
STAT: Signal transducer and activator of transcription
VSMC: Vascular smooth muscle cell
WB: Western blot
