# Supplementary material for: A miR-125/Sirtuin-7 pathway drives the pro-calcific potential of myeloid cells in diabetic vascular disease
Source: Diabetologia. 2022 Jun 16;65(9):1555–68. doi: 10.1007/s00125-022-05733-2 (PMC9345831; doi:10.1007/s00125-022-05733-2)
Supplement: Supplementary file 1 — (PDF 180 kb) [file 125_2022_5733_MOESM1_ESM.pdf]

Electronic Supplementary Material

ESM Methods

Coronary calcium quantification Coronary calcification (CC) was detected by coronary angiography. This methodology has a high predictive value for CC detection, although with low/moderate sensitivity [1]. Coronary angiography was performed using the standard technique by percutaneous femoral approach. Coronary stenosis was quantified according to Ambrose's method [2]. The angiographic classification was similar to that proposed by the American Heart Association (AHA). CC was detected by fluoroscopy, before contrast injection, as previously described [3]. Videotape recording allowed off-line analysis. Calcium content and stenosis were scored (diffuse; wide; long; spotty; none) in every single segment. The presence or absence of significant obstruction corresponding to calcification site was also evaluated, along with the number of vessels (0-3) and segments with calcification (n 0-9). The Syntax score was calculated for each patient.

Gene expression in human peripheral blood mononuclear cells (PBMCs) Total RNA was isolated from cells by RNeasy Mini kit (Qiagen, Hilden, Germany. For gene expression, cDNA was synthesized with 500 ng of RNA extracted using iScript cDNA synthesis kit (Bio-Rad, Hercules, CA) according to the manufacturer's instructions. qPCR was performed in a Bio-Rad CFX96 Real Time PCR detection system. Primers were designed from sequences derived from the GenBank database using Primer 3 (Whitehead Institute, Massachusetts, USA) and Operon's Oligo software (Operon, California, USA) and were purchased from Eurofins MWG (Ebersberg, Germany). The specificity of qPCR assay was validated by melt-curve analysis and agarose gel analysis. β-actin was used as reference gene. Data analyses were performed with the Bio-Rad CFX Manager. *BMP2, OC N, MGP, RUNX2, MSX2*, and *SP7* (*OSX*) gene or protein expressions were determined as described above. The specific primers are reported in ESM Table 1. When deemed appropriate, the comparative cycle threshold method (ΔΔCt), which compares the difference between groups in cycle threshold values, was used to obtain the relative fold change of gene expression. In independent experiments, Monocytes (upper layer) and lymphocytes (bottom layer) were separated by Ficoll® 1077 (Merck) processed cells by density gradient centrifugation for 15 minutes at 1700 rpm.

Release of paracrine factors THP-1 cells were cultured in 24-well plates in the presence and in the absence of osteogenic medium. Then, the culture medium was replaced with serum- free RPMI containing 1% FBS overnight. Concentrations of allograft Inflammatory Factor-1 (AIF-1) and S100 Calcium Binding Protein A8 (S100A8) were determined in the medium by ELISA (Elabscience Biotechnology Inc., Tema Ricerca, Bologna, Italy).

Western blot Cells were lysed in RIPA buffer, including protease inhibitors. Cell lysates (30 μg) were separated by SDS-PAGE using a 10% polyacrylamide gel. Then, proteins were electroblotted onto

nitrocellulose membranes (Hybond ECL, Amersham, UK), using a Transblot cell system (Elettrofor, Padova, Italy). The membranes were blocked overnight at 4°C in nonfat dry milk. Then, membranes were exposed to antibodies overnight at 4°C. The membranes were washed (4 times, 20 min each) in T-PBS and then incubated with horseradish peroxidase-conjugated secondary antibody (Cell Signaling Technology, ca# 7074; dilution 1:2000). Protein bands were visualized using an enhanced chemiluminescence detection system (Pierce Biotechnology, Rockford, IL). The blots were scanned and quantified using a chemiluminescence molecular imaging system (Bio-Rad, Hercules, CA). The results were expressed as densitometric evaluations normalized to GADPH, used as internal control. The protein content was determined by BCA reagents (Pierce), using bovine serum albumin as the standard.

RUNX2 acetylation RUNX2 lysine acetylation was analyzed by immunoprecipitation of RUNX2 followed by WB using acetyl-lysine antibody. hMSC grown in osteogenic medium were transfected with 100 nM siRNA against SIRT7 or with pcDNA3.1 SIRT7flag. Cells were lysed on ice by resuspension in ice-cold lysis buffer (50 mM Tris-HCl, pH 8.0, 150 mM NaCl, 10% glycerol, 0.5% Triton X-100, 25 mM glycerolphoshate, and protease inhibitors). After sonication, lysates were precleared by 30-min incubations with 100 µl of a 50% (v/v) suspension of Protein A-Sepharose beads coated with antibody against RUNX2 (Cell Signaling Technology, cat#. 8486) or with control IgG (Santa Cruz Biotechnology, cat# sc-69786). The beads were washed five times with buffer (50 mM Tris/HCl, pH 7.5, 150 mM NaCl, 1 mM MgCl2 and 0.05% Nonidet P40). Proteins were eluted from the beads by boiling in 2x Laemmli sample buffer for 5 min, resolved by SDS-PAGE 10%, and immunoblotted against anti-acetylated-lysine antibody (Cell Signaling Technology, cat# 9814).

Human telomerase reverse transcriptase (hTERT) expression hTERT expression in PMBCs was determined by qPCR, using specific primers TaqMan GeneExpression Assays for hTERT (Hs00972656_m1) and GAPDH (Hs03929097_g1), respectively. qPCR was performed in a Bio-Rad CFX96 Real Time PCR detection system. The reaction was performed in a 20 µl final reaction volume containing 200 nmol of each primer and SsoFast EVAGreen SuperMix (Bio-Rad, USA). Data were analyzed using Bio-Rad CFX Manager. The comparative cycle threshold method (ΔΔCq), which compares the difference between groups in cycle threshold values, was used to obtain the relative fold change of hTETR gene expression.

Telomere length Genomic DNA was extracted from the blood of 87 subjects with the EuroGold Blood kit (EuroClone, Italy). Mean telomere length was measured from DNA by a real-time PCR technique that compares the telomere (T) repeat sequence copy number to the single copy gene (S) in a given sample, as previously described [4]. Duplicate DNA samples were amplified in 25-µL PCR reactions composed of 15-ng genomic DNA, SsoFast EVAGreen SuperMix (Bio-Rad) and either 100 nmol/L of telomere-specific primers (forward: 5′-GGGTTTGTTTGGGTTTGGGTTTGGGTTTGGGTTTGGGTT-3′; reverse: 5′-GGCTTGCCTTACCCTTACCCTTACCCTTACCCTTACCCT-3′) or 100 nmol/L of the 36B4 forward

primer (5′-CAGCAAGTGGGAAGGTGTAATCC-3′) primer and 36B4 reverse primer (5′-CCCATTCTATCATCAACGGGTACAA-3′). Reactions were run on a Bio-Rad CFX96 detection system. Data were analyzed using the comparative Ct method (ΔΔCt) to calculate relative differences in the amount of telomere repeat copy number (T) to single-copy gene copy number (36B4 gene, S), with all samples being compared with the same reference, a pooled sample from multiple individuals.

Von Kossa Staining To verify calcification in cell cultures, we performed the von Kossa stain in hMSC and THP-1 osteogenic-differentiated cells. Differentiated-osteogenic cells were fixed with 4% paraformaldehyde for 15 mins at room temperature. The fixed cells were gently washed 3 times with distilled water and then stained with 1% silver nitride staining (Sigma-Aldrich) under UV radiation for 20 to 30 mins at room temperature. The stained cells were washed 2-3 times with distilled water, the unreacted silver was removed with 5% sodium thiosulfate for 5 minutes, and then were rinsed and kept in distilled water. The presence of black stain confirmed the presence of calcium phosphate deposition. The slides were counterstained with hematoxylin. Images were acquired using a Leica DMRE digital camera. ImageJ software was used to quantify the positive stain.

Quantitative calcium measure Calcium deposition was quantified based on a previously described method [5]. Cells were washed twice with phosphate buffered saline (PBS) and decalcified with 0.6 M HCl at room temperature for 2h. Free calcium was determined with a colorimetric assay by a stable interaction with phenolsulphonphthalein, using a commercially available kit (Chema Diagnostica. Monsano (AN) Italy), and corrected for total protein concentration (Pierce Biotechnology, Rockford, Illinois, USA), following solubilisation with 0.1 M NaOH/0.1% SDS. Absorbances were measured using a Mithras LB 940 Multimode Microplate Reader (Berthold Technologies. Bad Wildbad, Germany) at 570 nm (calcium) and at 690 nm (protein).

***In silico* selection of candidate microRNA** The selection of hyperglycaemia and osteogenic differentiation miRNA expression profiling datasets was performed using the publicly available GEO DataSets database. Briefly, for the selection of hyperglycaemia miRNA datasets the following search terms were used: ((“non-coding RNA profiling by array” (DataSet Type) AND “hyperglycaemia) AND “*Homo sapiens*”)); while for the selection of osteoblast differentiation BMSc datasets the search terms used were as follows: ((“non-coding RNA profiling by array” (DataSet Type)) AND BMSc) AND “*Homo sapiens*”). These search criteria allowed the identification of different miRNA expression datasets, which were further analysed using the GEO2R tool, and for the merging analyses through a Venn diagram calculating tool. Furthermore, we used another tool (http://mirwalk.umm.uni-heidelberg.de/) to identify those miRNAs which are possible targets for SIRT7.

miRNA gene expression *RNA Extraction*. microRNA from osteo-differentiated THP-1 cells was extracted using the miRNeasy mini kit (Qiagen), according to the manufacturer's protocol. *miRNA gene expression.* miR-125b, miR122, miR-93, miR-340, miR-34c-5p and miR-324-3p expression were detected by Taqman Advanced miRNA assay (Thermo Fisher). To normalise qPCR data, the RNU48 snRNA was used to normalize miRNAs expression, as previously described [6]. Data analyses were performed with the Bio-Rad CFX Manager. The comparative cycle threshold method (ΔΔCq), which compares the difference between groups in cycle threshold values, was used to obtain the relative fold change of gene expression.

RNA-sequencing analysis *RNA isolation***.** Total RNA was isolated from THP-1 cells grown in osteogenic medium for 21 days under normal (5 mM) or high (20 mM) glucose. The concentration and quality of RNA were measured by Nanodrop and Qubit 4.0. RNA integrity was further verified using LabChip Perkin Elmer. For each sample, 200 ng of RNA was used for RNA-seq library preparation using the Illumina Stranded mRNA prep. (Illumina, USA). Briefly, purified mRNA was fragmented using oligo (dT) magnetic beads to capture messenger RNAs (mRNAs) with polyA tails. mRNAs were copied into first-strand complementary (cDNA) and second-strand cDNA synthesis. Then, adenine / thymine and ligate adapters were added at the fragment ends. The resulting products were amplified to add indexes and primer sequences for cluster generation. A pool of libraries (1.2 pM) mix with Phix 2% was sequenced with a High-Output flow cell (75x2 cycles, up to 400 M of reads) using NextSeq 550 (Illumina, CA, USA). The number of reads for sample was ranged about 30-40 million. *Bioinformatic analysis*. Data analysis was performed using CLC Genomics Workbench software v.20 (Quiagen). Reads from FASTq files were filtered for quality and aligned to the Ensembl-v99-hg38 version of the reference human genome. The data were normalised using the TMM method (trimmed Mean of *M*-values). Statistically differentially expressed (DEG) transcripts were performed using Empirical analysis of Differential Gene Expression (EDGE, a count-based statistics) with expression values. Adjusted *p*-value of false discovery rate (FDR) was considered significant at <0.05. Principal component analysis (PCA) was represented in a two-dimensional plot to investigate the clustering of data. Volcano plot was generated setting the threshold for the Log_2 (Fold Change) to 2 and $-Log_{10}$(p-value) to 1.3 ($p<0.05$). Gene ontology (GO) and pathway enrichment analysis of significant differentially expressed genes were conducted using DAVID (Database for Annotation, Visualization, and Integrated Discovery, david.abcc.ncifcrf.gov) [7].

ESM References

[1] Andrews J, Psaltis PJ, Bartolo BAD, Nicholls SJ, Puri R (2018) Coronary arterial calcification: A review of mechanisms, promoters and imaging. Trends Cardiovasc Med 28: 491-501

[2] Ambrose JA, Winters SL, Arora RR, et al. (1985) Coronary angiographic morphology in myocardial infarction: a link between the pathogenesis of unstable angina and myocardial infarction. J Am Coll Cardiol 6: 1233-1238

[3] Bartel AG, Chen JT, Peter RH, Behar VS, Kong Y, Lester RG (1974) The significance of coronary calcification detected by fluoroscopy. A report of 360 patients. Circulation 49: 1247-1253

[4] Cawthon RM (2002) Telomere measurement by quantitative PCR. Nucleic Acids Res 30: e47

[5] Tsang HG, Cui L, Farquharson C, Corcoran BM, Summers KM, Macrae VE (2018) Exploiting novel valve interstitial cell lines to study calcific aortic valve disease. Mol Med Rep 17: 2100-2106

[6] Giannella A, Radu CM, Franco L, et al. (2017) Circulating levels and characterization of microparticles in patients with different degrees of glucose tolerance. Cardiovasc Diabetol 16: 118

[7] Dennis G, Jr., Sherman BT, Hosack DA, et al. (2003) DAVID: Database for Annotation, Visualization, and Integrated Discovery. Genome Biol 4: P3

ESM Table 1 List and sequence or primers used to analyse gene expression

**Gene**	**For Sequence**	**Rev Sequence**
SIRT1	TACCGAGATAACCTTCTGTTCG	ATGAAACAGACACCCCAGCTC
SIRT2	AGAAGCAGACATGGACTTCCT	CTCCCACCAAACAGATGAC
SIRT3	CTTGAGAGAGTGTCGGGCAT	AGAACACAATGTCGGGCTTC
SIRT4	TGGGATCATCCTTGCAGGTAT	TGGTCAGCATGGGTCTATCA
SIRT5	GCCAAGTTCAAGTATGGCAGA	CGCCGGTAGTGGTAGAA
SIRT6	ACCAAGCACGACCGCCAT	GGGTGGGAGATTCCTCCTT
SIRT7	ATGAGCAGAAGCTGGTGC	CTGTCTGGTGTCTGTGGA
NAMPT	CCAAGAGACTGCTGGCAT	CTGCTGACCACAGATACAGG
p53	AGCACTAAGCGAGCACT	TGAGTCAGGCCCTTCTGT
p66shc	AATCAGAGAGCCTGCCACATT	CTCTTCCTCCTCCTCATC
mTOR	CCTTCTGCCTTCACAGATACC	CATTGCCTTCTGCCTCTTATG
FOXO3	AAGCAGACCCTCAAACTGACA	CTCACGGTGTGCTCTGAAT
TLR2	TGATGCTGCCATTCTCATTC	CGCAGCTCTCAGATTTACCC
TLR4	TATCACGGAGGTGGTTCCT	TCAGGGGATTAAAGCTCAGGT
MSX2	GCCGCCGCCAAGACATA	TCTGCCTCCTGCAGTCTTT
MGP	CGGTAGTAACTTTGTGTTATGAATC	AGCTCGTGGACAGGCTTAGA
BMP2	CAGCTTCCACCATGAAGAATCT	CGAGTTGGCTGTTGCAGGT
Osteocalcin	AGGTGCAGCCTTTGTGTCC	TCAGCCAACTCGTCACAGTC
Osterix/Sp7	CCACCCATTCTTCAGGAGGT	CCTAATATCCCCAGCCCCAG
β-Actin	AGAGCTACGAGCTGCCTGAC	GGATGCCACAGGACTCCA

ESM Table 2 Expression of a selected range of longevity-associated markers in patients without (CC-) and with (CC+) coronary calcification. Data are presented as mean ± standard deviation

**Parameter**	**All (n = 87)**	**CC- (n = 18)**	**CC+ (n = 69)**	**p-value**
SIRT1 Gene	1.24±1.31	1.92±1.77	1.08±1.12	0.017
SIRT1 Protein	1.09±0.46	1.27±10,14	0.90±0.10	0.047
SIRT2 Gene	1.18±0.59	1.30±0.85	1.15±0.51	0.349
SIRT3 Gene	1.14±0.46	1.09±0.46	1.16±0.47	0.610
SIRT4 Gene	1.14±0.61	1.33±0.78	1.09±0.56	0.234
SIRT5 Gene	1.23±0.85	1.46±1.04	1.18±0.79	0.223
SIRT6 Gene	1.19±0.64	1.31±0.97	1.16±0.53	0.379
SIRT7 Gene	1.09±0.45	2.01±1.74	0.92±0.38	0.021
Sirt7 Protein	1.05±0.70	0.80±0.23	0.32±0.09	0.012
NAD activity	1.90±0.60	2.19±0.84	1.83±0.50	0.114
NAMPT Gene	1.73±2.16	1.26±0.83	1.91±2.43	0.287
p66shc Gene	1.10±0.35	1.00±0.30	1.12±0.36	0.238
p53 Gene	1.22±0.60	1.30±0.77	1.20±0.56	0.607
mTOR Gene	1.12±0.44	0.98±0.64	1.15±0.38	0.303
FOXO Gene	1.22±0.64	1.13±0.35	1.24±0.70	0.379
TLR4 Gene	1.09±0.39	1.02±0.37	1.10±0.40	0.472
TLR2 Gene	1.13±0.45	1.14±0.62	1.13±0.40	0.936

ESM Table 3 Differentially expressed genes RNA-seq was performed on osteo-THP-1 cultured in high (20 mM) glucose compared to normal (5 mM) glucose.

Genes upregulated in high versus normal glucose:

Gene symbol	Gene	Log fold change	Log p-value
TIPRL	TOR Signaling Pathway Regulator	2,54	6,46
RDX	Radixin	2,07	5,04
PPP1CC	Protein Phosphatase 1 Catalytic Subunit Gamma	1,73	4,99
STAT1	Signal Transducer And Activator Of Transcription 1	2,21	4,88
S100A8	S100 Calcium Binding Protein A8	3,45	4,80
EEF1E1	Eukaryotic Translation Elongation Factor 1 Epsilon 1	2,69	4,44
RPS3	Ribosomal Protein S3	1,40	4,41
S100A9	S100 Calcium Binding Protein A9	1,32	4,41
NR3C1	Nuclear Receptor Subfamily 3 Group C Member 1	1,85	4,38
ZC3H13	Zinc Finger CCCH-Type Containing 13	1,75	4,13
PTPN11	Protein Tyrosine Phosphatase Non-Receptor Type 11	2,80	4,12
PPCS	Phosphopantothenoylcysteine Synthetase	2,50	4,12
DHRS9	Dehydrogenase/Reductase 9	1,21	3,91
POMT1	Protein O-mannosyltransferase 1	1,36	3,54
GABPB1	GA Binding Protein Transcription Factor Subunit Beta 1	2,60	3,31
Runx2	RUNX Family Transcription Factor 2	2,50	3,31
STT3A	STT3 Oligosaccharyltransferase Complex Catalytic Subunit A	1,79	2,56
STAT3	Signal Transducer And Activator Of Transcription 3	2,25	2,34
S100P	S100 Calcium Binding Protein P	1,28	2,34
NIPAL2	NIPA Like Domain Containing 2	1,89	2,25
AIF1	Allograft Inflammatory Factor 1	1,64	2,24
THOC6	THO Complex 6	2,01	2,15
GAS5	Growth Arrest Specific 5	1,19	2,10
ROMO1	Reactive Oxygen Species Modulator 1	1,42	2,02
RN7SK	RNA Component Of 7SK Nuclear Ribonucleoprotein	1,29	1,97
MTFR1	Mitochondrial Fission Regulator 1	1,78	1,94
SENP5	SUMO Specific Peptidase 5	1,96	1,88
PUF60	Poly(U) Binding Splicing Factor 60	1,60	1,64
RPS9	Ribosomal Protein S9	1,07	1,62
SACM1L	SAC1 Like Phosphatidylinositide Phosphatase	1,39	1,61
sp7	Sp7 Transcription Factor	1,20	1,61

KHDRBS	RNA binding protein	1,55	1,60
PPP2R1B	Protein Phosphatase 2 Scaffold Subunit Abeta	2,35	1,49
RPP40	Ribonuclease P/MRP Subunit P40	2,02	1,44
ARPP19	CAMP Regulated Phosphoprotein 19	1,69	1,36
NBR1	NBR1 Autophagy Cargo Receptor	1,57	1,34

Genes down-regulated in high versus normal glucose:

Gene symbol	Gene	Log fold change	Log p-value
EGR1	Epidermal Growth Factor Receptor	-1,73	4,85
UBE3A	Ubiquitin Protein Ligase E3A	-1,43	4,64
PCMT1	Protein-L-Isoaspartate (D-Aspartate) O-Methyltransferase	-1,35	4,54
PTBP3	Polypyrimidine Tract Binding Protein 3	-3,10	4,41
MAZ	MYC Associated Zinc Finger Protein	-3,10	4,40
MBNL1	Muscleblind Like Splicing Regulator 1	-1,25	4,28
PPP4R3B	Protein Phosphatase 4 Regulatory Subunit 3B	-1,53	4,26
CLPTM1	CLPTM1 Regulator Of GABA Type A Receptor Forward Trafficking	-1,23	3,84
ARHGAP11A	Rho GTPase Activating Protein 11A	-2,03	3,62
ZNF518A	Zinc Finger Protein 518A	-2,24	3,59
KLF5	Kruppel Like Factor 5	-2,58	3,50
POU2F1	POU Class 2 Homeobox 1	-1,49	3,20
ERAP1	Endoplasmic Reticulum Aminopeptidase 1	-1,22	3,15
ATP6V0C	ATPase H+ Transporting V0 Subunit C	-2,40	3,00
UCHL5	Ubiquitin C-Terminal Hydrolase L5	-1,02	3,00
NIPA2	NIPA Magnesium Transporter 2	-1,19	2,93
ZMYM2	Zinc Finger MYM-Type Containing 2	-1,80	2,77
RLIM	Ring Finger Protein, LIM Domain Interacting	-1,36	2,56
COX11	Cytochrome C Oxidase Copper Chaperone COX11	-1,50	2,53
UBE2W	Ubiquitin Conjugating Enzyme E2 W	-1,54	2,35
SIRT7	Sirtuin 7	-1,98	2,33
PDK4	Pyruvate Dehydrogenase Kinase 4	-1,47	2,25
EIF4E	Eukaryotic Translation Initiation Factor 4E	-1,20	1,59
CHEK1	Checkpoint Kinase 1	-1,25	1,53
LIN9	Lin-9 DREAM MuvB Core Complex Component	-1,17	1,33
PPM1B	Protein Phosphatase, Mg2+/Mn2+ Dependent 1B	-1,09	1,33
USP1	Ubiquitin Specific Peptidase 1	-2,18	1,32

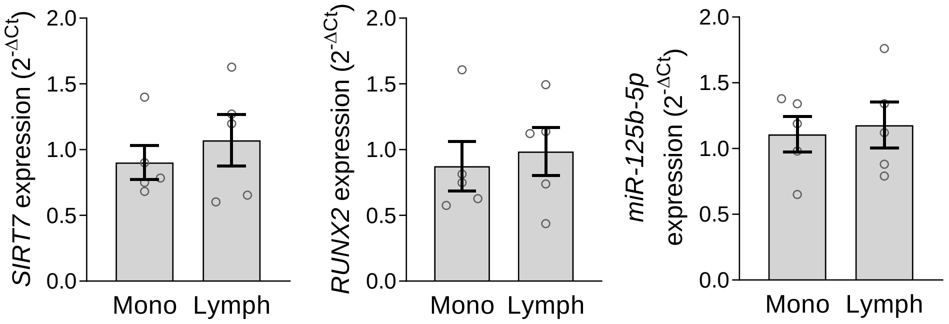

ESM Fig. 1 Gene expression in monocytes versus lymphocytes The mononuclear cell population was separated into monocytes and lymphocytes as described in the method section. Gene expression of SIRT7, RUNX2 and miR-125b-5p was analysed in the two sub-fractions and compared. No significant difference was detected. Histograms show means with standard error bars and superimposed circles indicate replicate experiments.

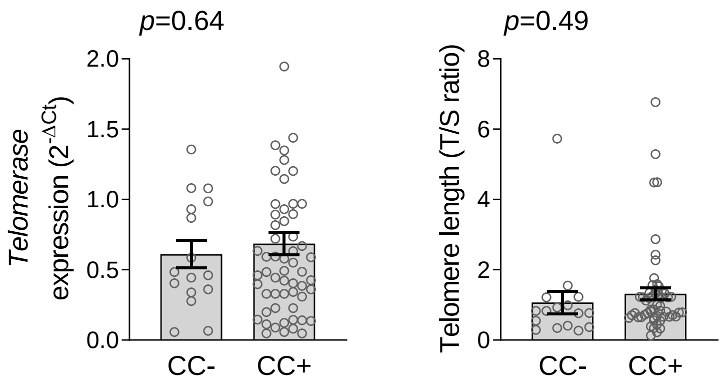

ESM Fig. 2 Telomere length and telomerase expression Gene expression of telomerase (left) and length of telomeres (right) was determined in mononuclear cells of patients with (+) or without (-) coronary calcification (CC). No significant difference was detected. Histograms show means with standard error bars and superimposed circles indicate replicate experiments.

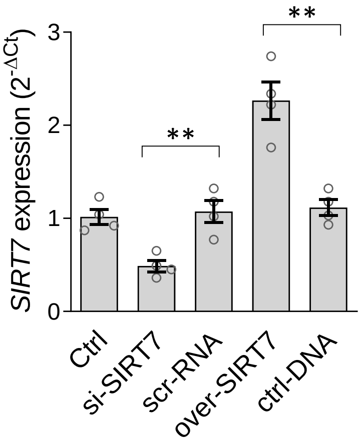

ESM Fig. 3 Knockdown and overexpression of SIRT7 in THP-1 cells. SIRT7 gene expression was determined in THP-1 cells treated with short interfering (si) RNA against SIRT7 or with a plasmid encoding for SIRT7 driving overexpression (over-SIRT7). The respective negative controls are shown for scramble (scr)-RNA and control (ctrl) void plasmid. **$p<0.01$ for the indicated comparison. Histograms show means with standard error bars and superimposed circles indicate replicate experiments.

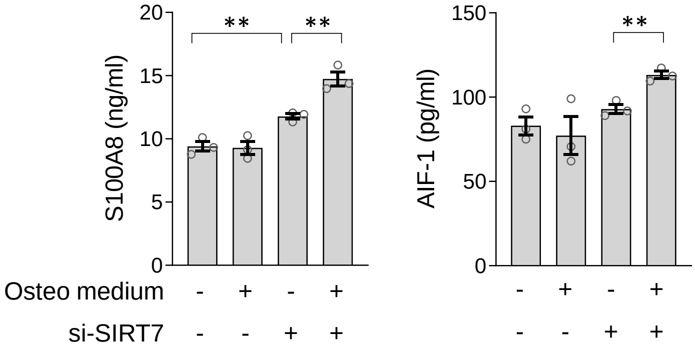

ESM Fig. 4 Pro-calcific factor secretion by osteo-induced THP-1 cells THP-1 cells were grown in control or osteogenic medium. After removing serum and incubation with RPMI, secreted S100A8 and allograft-inflammatory factor-1 (AIF-1) were measured by ELISA in the medium. **p<0.01 for the indicated comparison. Histograms show means with standard error bars and superimposed circles indicate replicate experiments.
